# Infant Feeding and Information Sources in Chilean Families Who Reported Baby-Led Weaning as a Complementary Feeding Method

**DOI:** 10.3390/nu13082707

**Published:** 2021-08-06

**Authors:** Daiana Quintiliano-Scarpelli, Nicole Lehmann, Bárbara Castillo, Estela Blanco

**Affiliations:** Carrera de Nutrición y Dietética, Facultad de Medicina-Clínica Alemana, Universidad del Desarrollo, Santiago 7610658, Chile; dquintiliano@udd.cl (D.Q.-S.); nlehmannb@udd.cl (N.L.); b.castillo@udd.cl (B.C.)

**Keywords:** baby-led weaning, complementary feeding, first foods, infants

## Abstract

Baby-Led Weaning (BLW) is a new and emerging method of introducing complementary feeding in solid consistency, without the use of a spoon and entirely guided by the baby. This study aims to analyze the implementation of the BLW approach in relation to compliance with basic components and sources of information in Chilean families. Using a cross-sectional design, we assessed early nutrition, including breastfeeding and foods offered, maternal/child characteristics and sources of information on BLW among a non-probabilistic sample of mothers of children <24 months who reported practicing BLW (*n* = 261, median age = 28 years) in Chile. We found that 57.5% of mothers reported their child ate the same food as the family, 44.1% shared ≥3 meals with the family, 84.7% offered ≥3 foods at each meal and 75.6% reported only occasionally offering food with a spoon. The majority reported obtaining information on BLW from social media (82%). Moreover, 56% had offered cookies, 32% added salt and 9% sugar in the first 2 years. Exclusive breastfeeding for 6 months related to higher odds of consuming family foods (OR = 2.45, 95% CI 1.24–4.84), while having received information from professional sources and social media related to lower odds (OR = 0.45, 95% CI 0.22–0.88 and OR = 0.31, 95% CI 0.15–0.66, respectively). Those who had appropriate weight gain had lower odds of consuming ≥3 foods in meals (OR = 0.35, 95% CI 0.13–0.96). Among mothers who reported practicing BLW with their children, we observed a wide variety of feeding habits, sources of information and low compliance with the studied components. Eating the same food as the family was the most prevalent component and social media was the main source of information on BLW.

## 1. Introduction

Traditionally, complementary feeding begins with the delivery of foods with the consistency of porridge or soft puree, without fibrous pieces to avoid stimulating the extrusion reflex, using a small smooth spoon delivered by a caregiver to the infant [1]. Baby-led weaning (BLW) is an approach for introducing solid food that allows the baby to self-feed at his/her own pace and decide how quickly to eat and how much to eat from the presented food [2]. BLW also refers to the inclusion of the infant into family mealtimes, where the infant eats the same food as the family, but in a suitable and safe form [3].

BLW may lead to improved feeding habits. The approach promotes autonomy, encourages the inclusion of infants in family meals and the consumption of healthy foods [4,5,6]. However, the potential increase in consumption of healthy foods for young children assumes that the family is eating healthy foods, which is not the reality for many around the world. In Chile, for example, despite recent positive changes in the food environment [7], very few adult Chileans meet the recommended healthy eating guidelines [8]. In addition, an increased risk in choking and nutritional deficiencies (e.g., zinc and iron) are common perceived consequences of traditional or modified BLW approaches, although that is not necessarily supported by evidence [9]. Another important challenge of adopting the method may be the lack of education on the part of health providers or hesitancy to recommend it.

Several studies have characterized samples that practice BLW, comparing them to those who practice more traditional complementary feeding practices, focusing primarily on maternal characteristics. Mothers who practice BLW tend to have a high level of education, exercise less control over food intake, use less emotional feeding and worry less about their child’s weight compared to their non-BLW counterparts [10,11,12]. Some evidence is also available on the first foods offered by families who practice BLW and their sources of information. One study reported that foods offered include fruits and vegetables, pastas and potatoes and that BLW families obtain information to guide feeding practices from the internet and social media [13].

In the last decade, there has been an increase in families around the world who decide to feed their children using the BLW approach. However, relatively little is known with respect to what elements of the approach are most followed among families who report BLW practices and what infant and maternal characteristics might relate to adherence. In addition, there is little information on families in Latin America that practice BLW [14]. Therefore, the objective of this study was to explore these factors (maternal education and working status, type of birth, breastfeeding, infant weight gain and sources of information on BLW) among a sample of Chilean mothers who self-identified as practicing BLW.

## 2. Materials and Methods

An exploratory cross-sectional study was carried out with a non-random sample of mothers of children younger than 24 months who self-reported having practiced BLW with their children who were born at term (≥37 weeks’ gestation) and residing in Chile. Data collection was conducted between August and December 2018. During this period, 364 mothers answered an online survey; 48 mothers were excluded because children were older than 24 months, 40 were excluded because the child was born preterm and 15 mothers did not live in Chile. Thus, a total of 261 participants were included.

Mothers were recruited from different social media groups (Facebook^®^, Instagram^®^ and Whatsapp^®^), information on BLW or other feeding practices was not delivered before participation. Educational material on BLW practices and recipes was sent after participation to those that provided their e-mail. Participants answered an online survey containing 47 questions, reviewed for internal validity by experts in pediatric nutrition. The survey included five sections: i. maternal sociodemographic information (e.g., age, nationality, educational level, profession and current occupation), ii. gestational and perinatal history (e.g., type of delivery, gestational age, birth weight of the child), iii. early nutrition aspects (e.g., duration of breastfeeding, first foods offered in the first two years of life), iv. sources of information on BLW (family/friends, nutritionist, pediatrician and/or midwife, social networks, television, books and others (more than one option could be selected) and v. adherence to components of BLW (described below). Most questions were asked retrospectively, for example: “what was the first food you offered to your child”. The survey was anonymous and voluntary, according to the principles of the Declaration of Helsinki. The study was reviewed and approved by the Ethics Committee of the Faculty of Medicine, Clinica Alemana, Universidad del Desarrollo, Santiago (PG 56/18).

For this study, we considered adherence to three components of BLW and one related to nutritional quality: (1) infant eats the same food as the family (reference = yes); (2) number of meals shared with the family members or caregivers (reference = 3 or more); (3) frequency of spoon use (reference = infrequently/never); and (4) number of foods (reference = 3 or more food groups) offered at a main meal (e.g., breakfast, lunch or dinner). In our study, offering at least three different food groups in a main meal was used to assess nutritional quality as part of a healthy food intake based on the Chilean recommendation for children under 2 [15].

### Statistical Analysis

Qualitative variables were presented using absolute frequency, while quantitative variables were expressed with median and interquartile range. Chi-square or Fisher’s exact tests were used to test the association between foods offered in the first 2 years and each component of BLW. Multivariate logistic regression was used to assess the association between maternal/infant characteristics (maternal education and working status, type of birth, breastfeeding in the first 6 months, adequate infant weight gain) and sources of information and each of the four BLW components using a stepwise forward procedure. The variables included in the multivariate model were those with *p* < 0.20 in the univariate model. All models were adjusted for the following covariates: maternal age and education level, independent significance level. In final multivariable models, covariates with *p* > 0.05 or multicollinearity (variance inflation factor > 4) were removed for parsimony. Goodness of fit was measured with the Hosmer–Lemeshow test. Alpha was set at *p* < 0.05. Statistical analysis was carried out using STATA 13.1 for Mac.

## 3. Results

Mothers had a median age of 28 years (IQR = 6 years), were Chilean (96.6%), reported being employed (68.2%) and 47.1% had more than 16 years of education. The majority (82.4%) of participants reported accessing information on BLW from social media, with 19% and 18% reporting having obtained information from health professionals and friends, respectively. 

Children were of average birth weight (91.9% between 2500 and 4000 g) and had been exclusively breastfed in the first 6 months of life (82.4%). During the practice of BLW, mothers reported that 78% of children experienced gagging, 28% choking and 3% suffocation. Additional information on characteristics of mothers and children is provided in Table 1. First food was given at a median age of 6.4 months (IQR = 0.7) and BLW began at a median age of 6.9 months (IQR = 1.0).

### 3.1. BLW Components

Table 2 provides a description of the four components of BLW evaluated in this study. With respect to sharing of family meals, 57.5% reported that their child ate the same food as the family and 44.1% participated in at least 3 family meals per day, with the lunch meal being the most frequently shared meal (52.9%). The majority (84.7%) were offered at least 3 different types of foods at each meal (breakfast, lunch or dinner) and were offered food with a spoon only occasionally or never (75.6%). In this sample, 19.2% (*n* = 50) adhered to all four components studied.

### 3.2. Foods Offered in the First 2 Years by BLW Component

Nearly half of the sample (46.7%) reported offering fruit as the first food. Seventy mothers (26.8%) reported offered vegetables, 30 (11.4%) offered cereals, 20 (7.6%) offered a fruit/vegetable mix, 15 (5.7%) soft puree and 4 (1.5%) mothers reported offering meat as the first food. Infants drank 2.6 (IQR = 2.7) servings of water and 1.6 (IQR = 1.7) servings of juice per day.

Table 3 provides details of foods offered in the first 2 years of life overall and by BLW component. All families reported having offered fruits and vegetables and the majority had offered cereals (94.6%), meat (86.6%), and fish/seafood (60.5%). Nearly one third of families (32.6%) reported having added salt to foods offered to infants, 8.8% added sugar and 7.7% offered honey in the first 2 years. Among families who offered juice in the first 2 years (19.9%), 32.1% of juice offered was natural. Very few families offered soda, coffee and tea to their children in the first 2 years.

Families who reported that infants consumed family foods were significantly more likely to have offered cookies in the first 2 years (63.5% vs. 46.9%), honey (11.3% vs. 2.7%) and fish/seafood (66% vs. 53.2%). They were also more likely to have added salt (38.7% vs. 24.3%) and sugar (13.3% vs. 2.7%), both *p* < 0.05. Families whose children ate at least 3 meals with the family were more likely to have been offered ham/sausage/salami com-pared to those who ate <3 meals with the family (13% vs. 4.8%, *p* < 0.05). These families were also significantly more likely to have offered cookies (64.4% vs. 50.0%) and fish/seafood (69.6% vs. 53.4%). Nearly 90% of families whose children consumed 3 or more foods during meals had offered meat, compared to 70% of families whose children consumed <3 foods during meals (*p* < 0.05). These families were also more likely to have offered cereal (96% vs. 85%, *p* < 0.05), cookies (60.6% vs. 32.5%, *p* < 0.05) and fish/seafood (66.1% vs. 30.0%, *p* < 0.05). We did not observe any statistically significant differences in the foods offered in the first 2 years when comparing families who only occasionally used a spoon versus those who used a spoon more frequently (Table 3).

### 3.3. Maternal Characteristics Related to Four Components of Baby-Led Weaning

Univariate and multi-variable logistic regression models are shown in Table 4. The results of the univariate and multi-variable analyses were virtually the same. After adjusting for maternal age and education level, we identified significant associations with 2 of the 4 components: consuming family meals and consuming ≥3 foods during meals. We identified 3 factors related to consuming family foods: breastfeeding, reporting having obtained information on BLW practices from a professional source and from social media. Specifically, exclusive breastfeeding up to 6 months related to higher odds of consuming family foods (OR = 2.45, 95% CI 1.24–4.84), while having received information from professional sources and social media related to lower odds (OR = 0.45, 95% CI 0.22–0.88 and OR = 0.31, 95% CI 0.15–0.66, respectively). For consuming ≥3 foods during meals, in the adjusted multivariable model, we found that those who had appropriate weight gain, compared to those who gained too little or too much weight between 6 and 12 months, had lower odds of consuming ≥3 foods in meals (OR = 0.35, 95% CI 0.13–0.96). We did not observe any statistically significant maternal or infant characteristics or reported sources of information on BLW practices that related to differential odds of eating ≥3 meals with the family per day or frequency of spoon use (Table 4).

## 4. Discussion

Our study provides evidence regarding how BLW is being practiced, considering maternal and infant characteristics, sources of information and eating habits among a sample of Chilean women, similar to samples evaluated in other contexts [16,17,18], who reported adhering, at least partially, to the practice. There is no single, gold-standard definition on what BLW practice should be. Based on the literature, we evaluated three BLW components (eating the same food as the family; sharing at least three meals with family/caregivers; infrequent spoon use) and a measure of meal quality (three or more foods offered at primary meals). We observed a variety of eating habits among families and, despite reporting using the BLW method, families had low adherence to the components evaluated. 

Our study showed that the most common source of information about BLW was social media. In Chile, the increasing popularity of the BLW practice is evident in social media; however, few health professionals have received formal training on the BLW approach, thus, as we reported here, information received by mothers seemed to come largely from informal and potentially non-scientific sources. A study conducted in 2019 among Chilean primary-care nutritionists revealed that 61.7% did not have the knowledge to guide parents about the BLW approach [19]. This may be one of the reasons that families reported using social media, and not health professionals, as sources of information on BLW practices. As health professionals begin to learn about BLW and how to implement the practice in accordance with local food guidelines, families may change their information-seeking practices. At minimum, parents could be informed, in a non-judgmental way, that there is a lack of robust evidence and standardized clinical guidelines with respect to BLW.

The introduction of complementary feeding is considered a window of opportunity for the establishment of healthy eating habits in childhood and into adulthood [20]. In our sample of families reporting the practice of BLW, we observed that the first foods offered were fruits (46%) and vegetables (26%). Our results are in agreement with other studies that similarly show similar proportion of preference for fruits and vegetables for the first offered foods [4,21]. We also identified several examples of non-age appropriate foods, liquids and additives being offered to infants according to Chilean feeding guidelines [15]. For example, we observed a high frequency of adding salt (32.0%), sugar (8.8%), honey (7.7%) or sweeteners to children’s food during the first two years of life. These habits are in conflict with national and international guidelines because they have been associated with poor dietary patterns [22]. Consumption of salt, sugar, sweetener and honey in children younger than 2 years of age has been associated with obesity and risk of botulism [23,24]. This is an important consideration for health professionals who have patients reporting BLW practice. While eating meals with the family is an important component of BLW, families should be reminded that foods should be offered to infants without additives. Public health efforts may need to focus on encouraging parents to adopt a healthy family diet prior to, and in preparation for infant weaning.

Previous studies have reported that in comparison with a traditional approach for the transition from liquid to solid food, the BLW approach appears to promote a healthier diet early in life, although not necessarily reducing risks of becoming overweight [20,25]. In our study we did not evaluate any other feeding approach, thus we were unable to compare between practices. We observed that families ate and shared with their infants ultra-processed foods like cookies and sausages during the first two years. These habits are similar to those reported in the results described by Rowan and Harris [26], who found that families did not change their eating habits before beginning BLW with their infants, which may have negative consequences for children. If families have poor nutritional habits, it is likely that these habits are passed on to infants during BLW, as one of the key components of the BLW practice is that the children eat the same food as the family if suitable and safe. In Chile, only 5% of the population has a healthy diet, measured by compliance to the National Food Guidelines [9]. Thus, it may not be surprising that participants reported that their children had also eaten these ultra-processed and unhealthy foods.

In our study, less than a quarter of the sample practiced the four components evaluated. A recent cross-sectional study conducted in Spain (Galicia, *n* = 6355 mothers) showed that full adherence to BLW is low among mothers who reported following this approach: 2.1% from an overall prevalence of BLW estimated at 14.0% [26]. Low adherence to BLW may be related to the lack of a standardized definition of this approach, which allows for considerable heterogeneity in families who self-identify as following BLW. We also noted that despite declaring the practice of BLW, many families fed infants with purees and using a spoon. Second, in many cases, families may have started BLW practices, but without access to a knowledgeable information source, they may mix eating practices.

Among the BLW components analyzed in this study, the component that appears to be the most difficult to accomplish was eating meals as a family. One study showed this may be due to the child having an earlier bedtime or to parental work commitments [27]. There are multiple benefits associated with the child eating during family meals, for example: learning the importance of waiting for one’s turn [28]. It is clear that the practice of BLW, specifically eating meals with the rest of the family, marks an initiation into the eating practices that continue for the rest of their lives.

Our multiple-variable models showed that exclusive breastfeeding and receiving information about BLW from social media were significantly associated with eating family foods. Particularly, longer breastfeeding was significantly associated with increased chances of eating the same foods as the family, on the contrary using social media as a source of information about BLW was associated with lower chances in practicing this component. In addition, breastfeeding was significantly associated with three of the four components (all but eating ≥3 meals with the family per day), but only reached statistical significance for eating the same foods as the family. It is possible, that the point estimate for eating ≥3 meals with the family per day would have reflected the direction demonstrated for the other three components. The precision of our confidence intervals should be improved in future studies with larger sample size.

The results of our study must be interpreted considering our limitations. First, participants were recruited via social media and the survey was completed online. This method, while fast and low-cost, may have introduced selection and information bias. For example, in this select sample, over 80% exclusively breastfed for at least 6 months, a rate much higher than that of the Chilean population [29]. However, our results are largely in agreement with other similar studies. All the information was self-reported by mothers, which may relate to information bias. Finally, as our study was retrospective there may be information bias in the form of difficulty remembering details of first foods among families. This might be especially true for mothers of older infants. In order to minimize bias, questions were presented with several possible alternatives, to help families remember. Our study focused on the first 2 years, future studies may consider sub-analyses by age of infant, as some habits may change over time (i.e., adding salt to foods may be more common among mothers of older infants). 

## 5. Conclusions

Our findings showed that, among families who self-reported practicing BLW, about 20% complied with the components of the BLW approach and nutritional quality measured. We observed that unhealthy foods were introduced to infants in the first 2 years of life and salt was added to foods in a third of the sample. BLW is a complementary feeding practice relatively new to Chilean families, which may also be the case in other contexts. More research is needed on pros/cons of the BLW approach in order to best orient health professionals and families. Despite the many benefits attributed to BLW, as observed in the current study, there may be considerable heterogeneity in eating habits among families practicing BLW.

## Figures and Tables

**Table 1:** Maternal and child characteristics and information sources on BLW practices.

Variables	%	n
Maternal characteristics		
Age, years *	28.0	6.1
Education		
<12 years	10.0	26
12–16 years	42.9	112
>16 years	47.1	123
Paid employment	68.2	178
Child characteristics		
Age, months *	12.8	7.5
Vaginal birth	52.1	136
Birth weight		
<2500 g	1.2	3
2500–4000 g	91.9	240
>4000 g	6.9	18
Female sex	52.1	136
Exclusive breastfeeding for up to 6 months	82.4	215
Food allergy	16.1	42
Appropriate growth between 6 and 12 months	75.9	198
Behaviors during BLW		
Gagging	78.2	191
Choking	28.4	74
Suffocation	3.1	8
Sources of information on BLW		
Health professionals	19.1	50
Social media	82.4	215
Family	5.8	15
Friends	18.4	48
Internet	11.9	31
Other	26.5	69

Abbreviations: BLW, baby-led weaning; IRQ, interquartile range; * data expressed in median and IQR.

**Table 2:** Description of components of baby-led weaning.

Variables	%	n
Consumes family foods	57.5	150
Shares ≥3 meals with the family per day	44.1	115
Which family meal shared		
Breakfast	4.2	11
Lunch	52.9	138
Snacks	18.4	48
Tea time	5.4	14
Dinner	13.8	36
All meals	44.1	115
Number of foods offered at main meals		
1–2	15.3	40
3–4	65.6	171
>4	19.1	50
Use of spoon		
Never	28.4	74
Occasionally (sometimes)	47.2	123
Frequently (at least once a day)	12.2	32
Always	12.2	32

**Table 3:** Type of foods offered in the first 2 years by BLW component adherence.

Food Groups ^1^	Overall	Consumes Family Foods		Eats ≥3 Meals with the Family		Consumes ≥3 Foods in a Main Meal		Does not Use a Spoon Frequently	
	*n =* 261	Yes (*n =* 150)	No (*n =* 111)	Yes (*n =* 115)	No (*n =* 146)	Yes (*n =* 221)	No (*n =* 40)	Yes (*n =* 197)	No (*n =* 64)
Soda	2.3 (6)	3.3 (5)	0.9 (1)	3.5 (4)	1.4 (2)	2.3 (5)	2.5 (1)	2.0 (2)	3.1 (4)
Coffee and tea	3.1 (8)	3.3 (5)	2.7 (3)	3.5 (4)	2.7 (4)	3.2 (7)	2.5 (1)	3.1 (6)	3.1 (2)
Meat	86.6 (226)	87.3 (131)	85.6 (95)	90.4 (104)	83.6 (122)	89.6 (198) ^†^	70.0 (28)	87.3 (172)	84.4 (54)
Cereals	94.6 (247)	96.0 (144)	92.8 (103)	97.4 (112)	92.5 (135)	96.4 (213) *	85.0 (34)	93.9 (185)	96.9 (62)
Candies	11.5 (30)	14.7 (22)	7.2 (8)	13.0 (15)	10.3 (15)	11.3 (25)	12.5 (5)	12.2 (24)	9.4 (6)
Ham/sausages	8.4 (22)	10.0 (15)	6.3 (7)	13.0 (15) *	4.8 (7)	8.6 (19)	7.5 (3)	7.1 (14)	12.5 (8)
Sweeteners	5.8 (15)	7.3 (11)	3.6 (4)	7.0 (8)	4.8 (7)	6.3 (14)	2.5 (1)	5.6 (11)	6.3 (4)
Fruits and vegetables	100 (261)	100 (150)	100 (111)	100 (115)	100 (146)	100 (221)	100 (40)	100 (197)	100 (64)
Cookies	56.3 (147)	63.3 (95) ^†^	46.9 (52)	64.4 (74) *	50.0 (73)	60.6 (134) *	32.5 (13)	55.3 (109)	59.4 (38)
Juices	19.9 (52)	21.3 (32)	18.0 (20)	21.7 (25)	18.4 (27)	19.0 (42)	25.0 (10)	20.3 (40)	18.8 (12)
Honey	7.7 (20)	11.3 (17) ^†^	2.7 (3)	11.3 (13)	4.8 (7)	8.6 (19)	2.5 (1)	7.1 (14)	9.4 (6)
Fish and seafood	60.5 (158)	66.0 (99) *	53.2 (59)	69.6 (80) ^†^	53.4 (78)	66.1 (146) ^†^	30.0 (12)	58.9 (116)	65.6 (42)
Salty snacks	13.4 (35)	16.7 (25)	9.0 (10)	16.5 (19)	11.0 (16)	13.1 (29)	15.0 (6)	12.7 (25)	15.6 (10)
Added salt	32.6 (85)	38.7 (58) *	24.3 (27)	34.8 (40)	30.8 (45)	34.0 (75)	25.0 (10)	30.0 (59)	40.6 (26)
Added sugar	8.8 (23)	13.3 (20) ^†^	2.7 (3)	10.4 (12)	7.5 (11)	9.5 (21)	5.0 (2)	8.1 (16)	10.9 (7)

^1^ % (n); Chi-square or Fisher Exact: * *p* < 0.05; ^†^ *p* < 0.01.

**Table 4:** Logistic regression models testing associations between maternal and infant characteristics, information sources on BLW and adherence to BLW components.

Variables	Consumes Family Foods		Eats ≥3 Meals with the Family per Day		Consumes ≥3 Foods in a Main Meal		Does not Use a Spoon Frequently	
	Univariate OR (95% CI)	Adjusted ^†^ OR (95% CI)	Univariate OR (95% CI)	Adjusted ^†^ OR (95% CI)	Univariate OR (95% CI)	Adjusted ^†^ OR (95% CI)	Univariate OR (95% CI)	Adjusted ^†^ OR (95% CI)
Mother reports paid work	0.68 (0.40–1.16)		0.97 (0.57–1.63)		0.68 (0.31–1.46)		1.68 (0.94–3.02)	
Vaginal birth	0.99 (0.61–1.62)		1.07 (0.66–1.74)		1.40 (0.71–2.75)		0.95 (0.54–1.67)	
Exclusive BF up to 6 months	2.22 (1.16–4.23)	2.45 (1.24–4.84)	0.64 (0.36–1.16)		1.71 (0.77–3.81)		1.11 (0.53–2.29)	
Appropriate infant growth	1.01 (0.57–1.81)		0.83 (0.47–1.46)		0.40 (0.15–1.07)	0.35 (0.13–0.96)	0.95 (0.49–1.85)	
Information sources								
Professionals	0.59 (0.31–1.12)	0.44 (0.22–0.88)	0.83 (0.44–1.58)		1.64 (0.61–4.44)		1.08 (0.51–2.27)	
Social media	0.41 (0.20–0.84)	0.31 (0.15–0.66)	1.43 (0.74–2.75)		0.99 (0.41–2.40)		1.64 (0.82–3.28)	
Family	0.84 (0.29–2.38)		1.48 (0.52–4.22)		0.71 (0.19–2.63)		1.32 (0.36–4.83)	
Friends	1.16 (0.61–2.20)		1.09 (0.58–2.05)		1.70 (0.63–4.57)		0.97 (0.47–2.00)	
Internet	2.45 (1.06–5.69)		1.51 (0.72–3.18)		2.98 (0.68–13.01)		0.97 (0.41–2.28)	
Other	1.62 (0.85–3.08)		1.41 (0.77–2.61)		2.43 (0.82–7.17)		1.07 (0.52–2.20)	

^†^ All models were adjusted for maternal age and education level. OR: odds ratio; CI: confidence interval; BF: breastfeeding.

## Data Availability

Not applicable.
